# Chronotype and Social Jetlag Influence Performance and Injury during Reserve Officers’ Training Corps Physical Training

**DOI:** 10.3390/ijerph192013644

**Published:** 2022-10-21

**Authors:** Graham R. McGinnis, Shani T. Thompson, Charli D. Aguilar, Michael B. Dial, Richard D. Tandy, Kara N. Radzak

**Affiliations:** Department of Kinesiology and Nutrition Sciences, University of Nevada Las Vegas, 4505 S. Maryland Pkwy, Las Vegas, NV 89154, USA

**Keywords:** social jetlag, chronotype, military training, sleep, musculoskeletal injury, Reserve Officers’ Training Corps

## Abstract

Sleep and circadian rhythms are critically important for optimal physical performance and maintaining health during training. Chronotype and altered sleep may modulate the response to exercise training, especially when performed at specific times/days, which may contribute to musculoskeletal injury. The purpose of this study was to determine if cadet characteristics (chronotype, sleep duration, and social jetlag) were associated with injury incidence and inflammation during physical training. Reserve Officers’ Training Corps (ROTC) cadets (*n* = 42) completed the Morningness/Eveningness Questionnaire to determine chronotype, and 1-week sleep logs to determine sleep duration and social jetlag. Salivary IL-6 was measured before and after the first and fourth exercise sessions during training. Prospective injury incidence was monitored over 14 weeks of training, and Army Physical Fitness Test scores were recorded at the conclusion. Chronotype, sleep duration, and social jetlag were assessed as independent factors impacting IL-6, injury incidence, and APFT scores using ANOVAs, chi-squared tests, and the *t*-test where appropriate, with significance accepted at *p* < 0.05. Evening chronotypes performed worse on the APFT (evening = 103.8 ± 59.8 vs. intermediate = 221.9 ± 40.3 vs. morning = 216.6 ± 43.6; *p* < 0.05), with no difference in injury incidence. Sleep duration did not significantly impact APFT score or injury incidence. Social jetlag was significantly higher in injured vs. uninjured cadets (2:40 ± 1:03 vs. 1:32 ± 55, *p* < 0.05). Exercise increased salivary IL-6, with no significant effects of chronotype, sleep duration, or social jetlag. Evening chronotypes and cadets with social jetlag display hampered performance during morning APFT. Social jetlag may be a behavioral biomarker for musculoskeletal injury risk, which requires further investigation.

## 1. Introduction

The importance of sleep and circadian rhythms in maintaining an individual’s health and achieving optimal physical performance continues to emerge. This relationship is particularly important for active individuals, such as athletes and military personnel, who perform high levels of training, often at various times throughout the day. Though peak exercise performance generally occurs in the afternoon [1], the specific time can be modulated by a person’s preference for morningness or eveningness (i.e., chronotype) [2]. Exercise training outside of the temporal window of peak performance, or at times of day misaligned with chronotypical preference, could be at the cost of performance. In fact, morning-type individuals display greater cognitive (via psychomotor vigilance tests) and physical performance (via maximal voluntary contraction) compared to evening types when tested in the morning. However, that effect is reversed when tests are performed later in the day, where evening types perform better [3]. Furthermore, high-intensity exercise performed in the morning was found to induce higher levels of stress (i.e., cortisol) in evening-type participants compared to morning types [4]. However, longitudinal studies evaluating the effects of training in line with chronotype are limited. As such, time-mandated physical exercise asynchronous to one’s chronotype could be maladaptive, and potentially increase musculoskeletal injury (MSKI) risk [5,6,7,8,9]. This represents a significant problem in the military in particular, as nearly 50 million limited duty days can be attributed to MSKI [10]. Understanding the factors associated with chronotype and sleep that impact the response to training is critical to maximize training efficacy and minimize maladaptive responses and injury.

A potential mechanism mediating the effects of chronotype on performance is sleep (e.g., evening-type individuals stay up later and get less sleep when forced to perform morning exercise). Decreased sleep duration has been found to negatively impact aerobic capacity and muscular endurance in both traditional athletic and military populations [11]. Furthermore, sleep deprivation has a profound negative impact on health, spanning cognitive performance, and mental and cardiometabolic health [12]. Poor sleep impairs cognitive and motor performance within the military population [13,14], and is associated with increased markers of inflammation, including interleukin-6 (IL-6) [15,16]. Recent findings support the hypothesis that sleep is a critical mediator of MSKI and recovery [17,18], bringing sleep and circadian rhythms to the fore in sports performance, military medicine, and public health [19]. 

Though sleep restriction and deprivation impair exercise performance, there is also an increase in the risk of musculoskeletal injury associated with poor sleep. In a cohort of high school athletes, sleeping less than 8 h per night was the best predictor of injury, associated with a 2.1-fold (95% CI, 1.2–3.9) increase in injury [20]. In addition, U.S. Army Special Forces Operators who sleep ≤ 4 h are at a 2.3-fold (95% CI, 1.9–2.9) greater risk for musculoskeletal injury when compared to peers who sleep ≥ 8 h [21]. Though sleep duration is one component of sleep quality, other factors likely play an important role. In support, poor sleep quality (based on the Pittsburgh Sleep Quality Index) has also been associated with increased odds for reporting a running-related injury (odds ratio = 1.24, 95% CI, 1.2–1.3) [22], as well as military-basic-training-related injury (odds ratio = 2.3, 95% CI, 1.6–3.4) [23]. 

More recently, a novel paradigm of circadian rhythm disruption has garnered increasing interest; social jetlag (SJL). SJL occurs when social clocks (i.e., free days) and work clocks (i.e., work days) are different, causing people with SJL to shift their behavior back and forth between work and free days [24]. These behaviors can include the timing of meals, sleep opportunities (staying up later, sleep in later on free days), as well as physical activity. Nearly 80% of the general population’s circadian sleep window is misaligned to their daily schedule [25], with ~85% experiencing some level of SJL [24]. Interestingly, in a cross-sectional analysis of adolescents, Higgins et al. found that cardiorespiratory fitness (i.e., VO2max) was lower in participants with greater SJL compared to those without [26]. Furthermore, epidemiological findings show that social jetlag is associated with other indices of poor metabolic health (high BMI) [27]. Importantly, the effects of SJL on BMI and cardiorespiratory fitness can be seen in the absence of sleep loss, providing strong evidence that the time-shifting of behaviors, not sleep loss, is the underlying cause [26,27]. Though research suggests a link between SJL, health, and performance, the relationship between SJL and MSKI has not, to our knowledge, been evaluated. 

Therefore, the purpose of the current study was to explore the relationship between sleep health and the response to early morning time-mandated training in ROTC cadets. We evaluated three parameters: (1) chronotype, (2) sleep duration (SD), and (3) social jetlag (SJL), in relationship to markers of musculoskeletal health, including injury incidence, inflammation, and maximal physical performance, over a semester of military training. We hypothesize that cadets with misaligned chronotypes, shorter SD, and greater SJL will have a maladaptive response to time-mandated morning physical training.

## 2. Materials and Methods

### 2.1. Participants 

A single university’s Army Reserve Officers’ Training Corps (ROTC) battalion was recruited to participate in the study (*n* = 42). The inclusion criteria were: (1) Army ROTC cadets, (2) ≥18 years of age, and <44 years of age. Cadets were excluded based on the presence of current musculoskeletal injury restricting participation in ROTC Physical Training (PT), chronic cardiovascular disease, respiratory disease, metabolic disease, and pregnancy or a plan to become pregnant during the study period. Participants were informed of the voluntary nature of the study, and provided written informed consent. All protocols were approved by the UNLV Institutional Review Board (#1294978).

### 2.2. ROTC Physical Training and Testing 

ROTC cadets participated in morning physical training (PT) Monday–Thursday from 06:00 h–07:00 h for 14 weeks. Each PT session varied in exercise composition, and included aerobic, strength, and power training activities designed to improve cadets’ physical fitness and prepare them for the Army Physical Fitness Test (APFT) [28]. The APFT includes scored performances on 2-min push-ups, 2-min sit-ups, and 2-mile run, and was performed at the end of the semester at 06:00. 

### 2.3. Baseline Data Collection 

Prior to the first training session, participants completed anthropometric assessments of height and weight using a calibrated scale and stadiometer. Chronotype was assessed using the Morningness/Eveningness Questionnaire (MEQ), and distributed into morning-type (M-Type), intermediate-type (I-Type), and evening-type (E-Type). Sleep duration (SD) and social jetlag (SJL) were assessed using the American Academy of Sleep Medicine’s Sleep Diary supplied at the baseline meeting, and were completed throughout the first 7–10 days of PT. Social jetlag (SJL), defined as the absolute difference between the mid-sleep phase of free days and work days, was calculated for each participant.

### 2.4. Saliva Collection and IL-6 ELISA

Saliva samples were collected via the passive drool technique prior to (PRE), and immediately following (POST) PT sessions on the first (D1) and fourth day (D4) of training, and stored at −80 °C. Saliva was used for the assessment of IL-6, a biomarker of inflammation. Participants were instructed to avoid eating and drinking for at least 30 min prior to each saliva collection. Participants that were unable to produce saliva during any of the collection periods were excluded from analysis at all time points. 

For IL-6 analysis, samples were thawed to room temperature and centrifuged at 1500× *g* for 15 min to clear the saliva of mucins. IL-6 was assessed in cleared saliva using a commercially available Enzyme-linked Immunosorbent Assay (ELISA; Salimetrics, State College, PA, USA) per the manufacturer’s instructions. Absorbance was measured at 450 nm with a secondary correction read at 620 nm, using the delta absorbance for analysis. In the case that any samples yielded absorbance too high for the spectrophotometer (indicating supramaximal IL-6 levels), the participant’s IL-6 data for all time points were excluded from statistical analysis. 

### 2.5. Injury Incidence and APFT Score

An embedded certified athletic trainer (AT) was present during all training sessions. The AT provided medical care for any injuries or complaints of the cadets, including the assessment of MSKI. Injury incidence was recorded using standard medical documentation throughout the training period, and included any instance a cadet sought medical attention from the AT. At the conclusion of the semester, a member of the research team performed a chart review of the participating cadets’ medical records to identify if the participating cadet sustained a MSKI (defined as any injury to the musculoskeletal system, including those with neurological involvement, which results in pain and a decrease or modification in training, regardless of whether training time was lost). 

### 2.6. Data and Statistical Analysis

To assess the impact of different sleep characteristics on inflammation and injury rates, we performed separate analyses for chronotype (participants grouped by MEQ scores), sleep duration (participants grouped by average SD), and social jetlag (participants grouped by the magnitude of SJL). Within each variable, APFT score was compared using 1-way ANOVA with Tukey post-hoc tests (for MEQ) or *t*-tests (for SD and SJL), whereas salivary IL-6 was compared using 2-way ANOVA. The injury incidence between groups was compared using a chi-squared test of independence. In the event that assumptions were violated, preventing the use of the Pearson’s chi-squared value, the likelihood ratio was used [29]. The difference in sleep characteristic values (i.e., MEQ score, SD, or SJL) between injured and uninjured cadets was compared using *t*-tests. Statistical analyses were performed using SPSS (Version 25.0, IBM, Armonk, NY, USA), and significance was accepted at *p* < 0.05.

## 3. Results

From the 42 cadets recruited, complete information was available for 27 cadets for the analysis of chronotype, and 19 cadets for the analysis of SD and SJL. The resulting cohort was majority-male (21 males, 6 females). The anthropometric data are seen in Table 1. 

### 3.1. Chronotype

**Figure 1:**
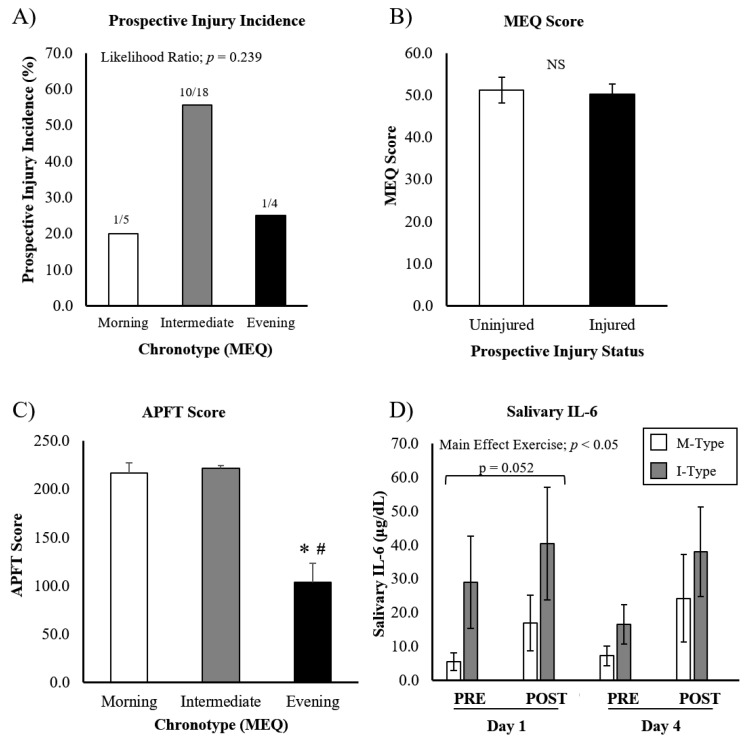
APFT performance, injury incidence, and IL-6 response to ROTC-PT based on chronotype. (**A**) Prospective injury incidence was recorded during the semester of ROTC PT, and was not different between chronotype groups. (**B**) Mean MEQ scores were not different between injured and uninjured cadets. (**C**) APFT test was performed at the end of training. (**D**) Salivary IL-6 increased in response to exercise, but was not different based on chronotype. Note: salivary IL-6 data for evening chronotypes are not presented, as all cadets in this group were above the detection limit of the assay, suggesting very high IL-6 levels in this group. Data are presented as mean ± SEM. * different from ‘M-Type’, ^#^ different from ‘I-Type’, *p* < 0.05.

Among the 27 cadets included in the analysis of chronotype, we found five M-Type, eighteen I-Type, and four E-Type participants. Injury incidence was assessed by evaluating the prevalence of injury within each chronotype (Figure 1A), as well as by comparing the raw MEQ score between uninjured and injured cadets (Figure 1B). A total of 12 cadets sustained an injury (out of 27): 1 morning-type, 10 intermediate-types, and 1 evening-type (Figure 1A). A chi-squared analysis revealed no significant differences in injury incidence between chronotypes (likelihood ratio; *p* = 0.24). Furthermore, the MEQ scores of cadets that sustained an injury throughout the training period were not different from cadets that remained uninjured (uninjured MEQ = 50.3 ± 2.5, injured MEQ = 51.2 ± 3.1; *p* = 0.41) (Figure 1B). E-Type cadets scored significantly lower on the APFT compared to I-Type and M-Type cadets (103.8 ± 34.5 vs. 221.9 ± 9.8 vs. 216.6 ± 21.8, respectively; *p* < 0.05), suggesting maximal exercise performance was hampered when not synchronized with circadian preferences (Figure 1C). 

A total of 20 participants with MEQ scores provided saliva for the analysis of IL-6 during exercise training (Figure 1D). We were unable to include E-Type cadets in this analysis, as all samples were above the detection limit of the ELISA. Accordingly, the effects of chronotype are reported between M-Type (*n* = 6) and I-Type (*n* = 14) cadets, where there was no difference between chronotypes (main effect chronotype; *p* = 0.33). A main effect for exercise was seen (*p* < 0.05), suggesting IL-6 increased in response to exercise. We additionally found a chronotype x day interaction (*p* = 0.02). Paired-sample *t*-tests revealed that the IL-6 levels on day 1 tended to be higher in I-Types compared to M-Types (*p* = 0.052), whereas no difference was seen on day 4. However, no significant interaction between chronotype x exercise was found, indicating exercise-induced IL-6 was not different between chronotypes. 

### 3.2. Sleep Duration

A total of 19 participants provided sleep diaries and completed the APFT. Cadets were separated into two groups based on their average sleep duration: SD ≤ 7:30 h (*n* = 8) or SD > 7:30 h (*n* = 11). 

**Figure 2:**
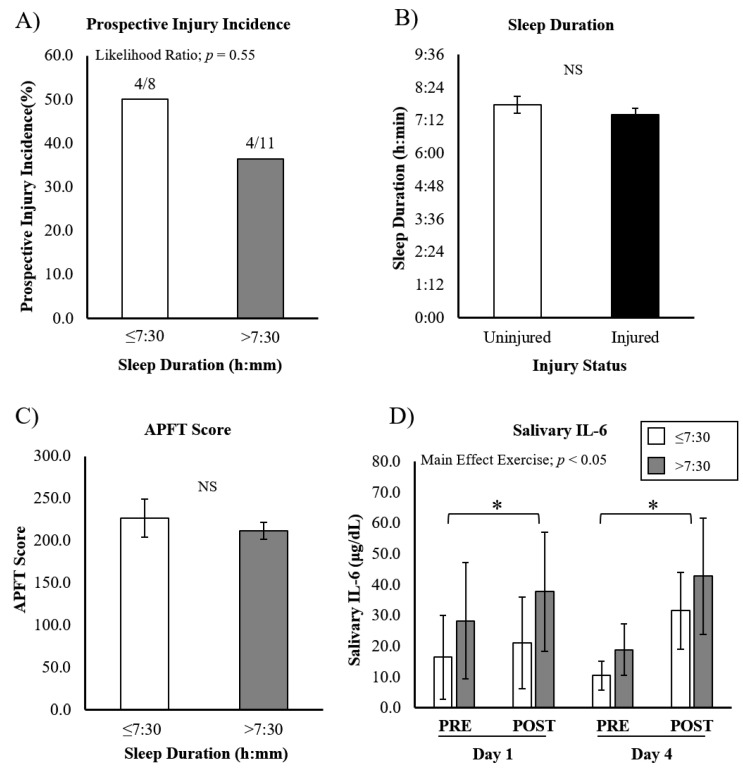
APFT performance, injury incidence, and IL-6 response to ROTC-PT based on sleep duration. (**A**) Prospective injury incidence was not significantly different between groups based on average self-reported sleep duration. (**B**) Average sleep duration was not significantly different between injured and uninjured cadets. (**C**) APFT score was not different between cadets with different sleep durations. (**D**) Salivary IL-6 increased in response to exercise, but was not different between cadets with different sleep durations, or on different days. Data are presented as mean ± SEM. * different from ‘PRE’.

We found that there was no difference in injury incidence between SD groups: 4/8 in SD ≤ 7:30 and 4/11 in SD > 7:30 (Figure 2A) (likelihood ratio; *p* = 0.552). Furthermore, the average SD was not different between injured and uninjured cadets (uninjured SD = 7:45 ± 0:59, injured SD = 7:24 ± 0:35; *p* = 0.17) (Figure 2B). No significant differences were seen between the SD categories on APFT scores (≤7:30 = 226.9 ± 22.5 vs. >7:30 = 211.9 ± 10.0; *p* = 0.49), indicating sleep duration did not impact maximal exercise performance (Figure 2C). 

From the 19 cadets used for SD analysis, saliva from 15 was assessed for IL-6 during training (*n* = 6 with SD ≤ 7:30, and *n* = 9 with SD > 7:30). A significant exercise x day interaction was seen (*p* < 0.05). However, post-hoc testing revealed that IL-6 was increased post-exercise on D1 and D4 (*p* < 0.05, both), with no significant differences between the two days. No significant differences were seen in exercise-induced salivary IL-6 between SD groups (exercise x SD interaction; *p* = 0.80), and the simple main effect of SD was also not significant (*p* = 0.74) (Figure 2D). 

### 3.3. Social Jetlag

**Figure 3:**
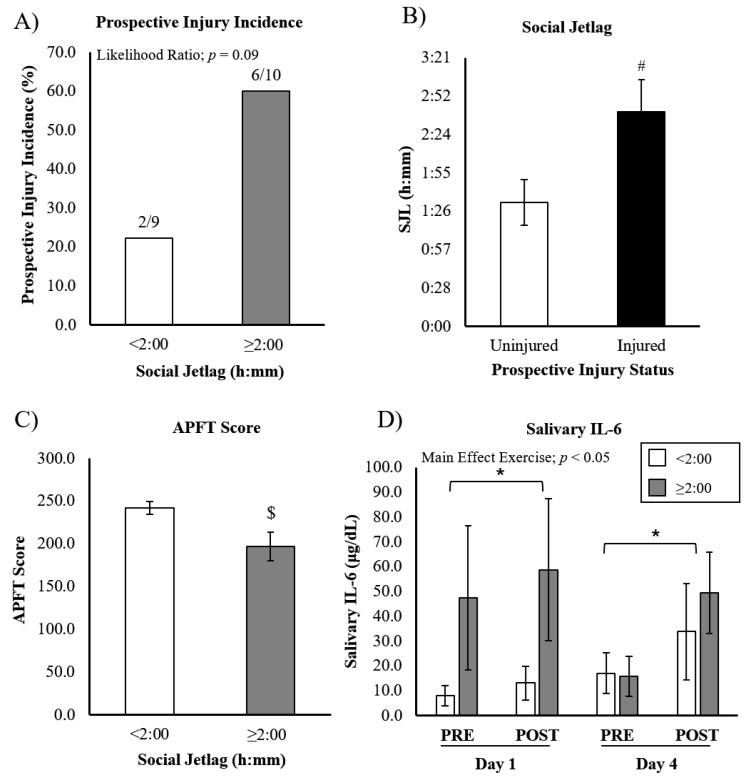
APFT performance, injury incidence, and IL-6 response to ROTC-PT based on social jetlag. (**A**) Injury incidence in cadets with <2:00 SJL or ≥2:00 SJL. (**B**) Cadets that sustained an injury reported significantly higher SJL compared to uninjured cadets. (**C**) APFT scores were significantly lower in cadets with >2:00 SJL. (**D**) Salivary IL-6 increased in response to exercise, but was not different between SJL groups, or between days. Data are presented as mean ± SEM. ^#^ different from ‘Uninjured’, ^$^ different from ‘SJL < 2:00’, * different from ‘PRE’, *p* < 0.05.

Social jetlag was assessed in a total of 19 participants, separated into two groups by the magnitude of SJL: SJL < 2:00 (*n* = 9) and SJL ≥ 2:00 (*n* = 10). The incidence of injury within SJL groups revealed two injuries for cadets with SJL < 2:00 (out of nine), and six injuries for cadets with SJL ≥ 2:00 (out of ten; Figure 3A). Chi-squared analysis revealed that this difference approached, but did not reach, statistical significance (likelihood ratio; *p* = 0.09). We subsequently calculated the average SJL in injured and uninjured cadets, revealing a significantly greater duration of SJL in injured cadets (injured SJL = 2:40 ± 0:24, uninjured SJL = 1:32 ± 0:17; *p* < 0.05) (Figure 3B). APFT scores were significantly lower in cadets with greater SJL (SJL < 2:00 = 241.9 ± 7.4 vs. SJL ≥ 2:00 = 196.9 ± 16.8; *p* < 0.05), suggesting cadets with greater SJL have compromised exercise performance (Figure 3C). 

The analysis of salivary IL-6 was performed on a total of 14 cadets (*n* = 8 with SJL < 2:00 and *n* = 6 with SJL ≥ 2:00) (Figure 3D). No significant differences were seen in IL-6 levels between different SJL groups (main effect SJL; *p* = 0.44), nor was exercise-induced IL-6 different between SJL groups (exercise x SJL interaction; *p* = 0.51). However, we again found an exercise x day interaction (*p* < 0.05), with IL-6 being increased post-exercise on D1 and D4, with no significant difference between days.

## 4. Discussion

The primary goal of this study was to determine if different sleep characteristics, including chronotype, sleep duration, and social jetlag, contributed to increased inflammation and injury incidence, or APFT scores, during early-morning time-mandated physical training in ROTC cadets. Our results support the hypothesis that E-Type cadets have hampered performance during early morning exercise, but without a substantial impact on injury incidence. Alternatively, SJL was identified as a novel contributing factor to impaired exercise performance and MSKI incidence, as cadets that sustained injury during the training period reported SJL at a significantly higher magnitude. To our knowledge, this is the first study to link SJL to MSKI. 

Previous studies have shown that athletic performance is influenced by chronotype, and that individuals may perform sub-optimally if forced to exercise at asynchronous times of day [3,30]. Furthermore, Lim et al. recently demonstrated lower peak power and greater fatigue in maximal effort Wingate tests in E-types even when performance times were matched to chronotype, suggesting compromised performance in E-types [31]. These results are similar to our findings that evening-type ROTC cadets performed significantly worse on the APFT compared to I-type and M-type cadets. Whether this is a product of hampered adaptation to a semester of training out of synchrony with temporal preference, or simply poor performance based on the time of the APFT (06:00 h), cannot be determined in this study. 

Though exercise performance was hampered in evening-type cadets, exercise-induced salivary IL-6 was not different between chronotypes (I-Type and M-Type). Though E-Type samples were not included in the analysis, we can conclude that they were extremely high, suggesting misaligned exercise was more pro-inflammatory in these participants. Similar to this assertion, Bonato et al. recently found that E-type participants had higher exercise-induced cortisol levels (a biomarker for stress) following a bout of morning exercise when compared to M-type participants [4]. Furthermore, it is interesting that I-Type IL-6 tended to be higher than M-Type on D1 (statistical trend), but similar on D4. This suggests that some adaptation or alignment may occur during the week as cadets adjust to early morning PT. Though our data did not statistically support a relationship between chronotype and injury, some controversy exists. Work-related MSKI was shown to be higher in E-type individuals in a large cohort of nurses [32], although participants were not participating in exercise training. Similar to our findings, Biggins et al. did not find a significant relationship between chronotype and self-reported injury in a population of elite university athletes [33]. Clearly, this relationship demands further consideration in subsequent studies.

Our data failed to identify a significant effect of sleep duration on injury incidence. These findings are in contrast to several recent studies investigating the relationship between sleep and musculoskeletal injury in athletes and military [21]. Grier et al. was able to show a significant increase in musculoskeletal injury rates associated with short sleep in a large cohort of Special Operations personnel (*n* > 7000) [21]. Interestingly, the magnitude of risk increased to a maximum effect for those with <4 h of sleep. This is a substantially shorter average sleep duration than was seen in our population, which may explain the discrepancy in findings. Likewise, Ruan et al., in a population of >500 military recruits, showed that poor sleep quality was associated with an increased incidence of injury [23]. However, with a sample size similar to ours, the relationship between sleep duration and injury incidence in a cohort of Australian footballers was not significant [34], supporting the need for a larger cohort in future studies. Additionally, the average sleep duration of our entire cohort was approximately 7.5 h, which is more than 1 h longer than a recent report on ROTC cadet sleep quality [14]. 

Insufficient sleep results in compromised athletic performance [11]. Interventional studies controlling for sleep duration have shown that subjective effort (rating of perceived exertion: RPE) during an exercise task progressively increased over subsequent days of sleep restriction (~3 h/night) [35]. Though this was shorter sleep than most of the participants in the current study, these results support the notion that 1 week, or an entire semester, of disrupted sleep via early morning PT may exacerbate the response to exercise. Sleep restriction has also been shown to exacerbate inflammatory biomarkers [15,36]. Cullen et al. showed that a single night of sleep deprivation increased resting levels of blood IL-6 levels, decreased performance, and increased RPE. No significant effects were seen during partial sleep deprivation, which may explain our equivocal findings in cadets with shorter sleep [15]. In sum, our data do not support a relationship between sleep duration and a pathophysiological response to ROTC PT.

Our study is the first to investigate the effects of SJL on MSKI. Previous observational studies of large cohorts have identified that SJL prevalence follows a normal distribution, with the average around 1 h SJL [24]. Our small cohort demonstrated slightly higher levels of SJL on average (~2 h), with only two cadets having <1 h SJL. This is actually similar to the average SJL reported for a population of night shift nurses [37], indicating rather extreme desynchrony between ROTC-related schedules and social clocks. Though several studies have found a relationship between SJL and chronotype (indicating evening types have a greater discrepancy between work and social clocks) [24,38], we observed weak associations between SJL and MEQ scores (R^2^ = 0.09), as well as SJL and SD (R^2^ < 0.01, data not shown). As such, we are interpreting our SJL results as independent of chronotype and SD.

Though previous studies have not investigated the effects of SJL on exercise training-induced adaptations, there have been observational studies (in adolescents) supporting the notion that individuals with higher SJL have lower levels of cardiorespiratory fitness [26]. Whether this is related to acute or chronic decrements in performance should be investigated further. In the present study, cadets with greater SJL (>2 h) had lower APFT scores. Furthermore, though we have not found any previous investigations linking SJL to MSKI, Umemura et al. determined that greater levels of SJL (determined via actigraphy) were associated with impairments in postural balance tasks [39]. When considering the physical demands of training, impaired balance could feasibly translate to injury risk. 

The results in this study suggest that the exercise training response is exacerbated when the training is misaligned with an individual’s chronotype (i.e., evening-type exercising in the early morning), and in people with SJL. Subsequent studies are needed to further substantiate these relationships in more tightly controlled clinical trials, with a wider evaluation of pro- and anti-inflammatory biomarkers, in order to evaluate the balance of exercise-induced hormesis [40]. Specifically, evaluating the training-induced adaptations and/or improvements in individuals with SJL vs. those without would allow more specific recommendations for athletes in training. Similarly, determining if chronotype is shifted during time-mandated training (adaptation), or if chronically misaligned training increases inflammation/stress (pathology), is warranted.

### Limitations

The current study was constrained by several limitations. Of specific importance was the limited sample size that was recruited and fully participated. A limited sample size, especially in regard to sleep logs and salivary IL-6, hampered our ability to detect statistically significant effects in several analyses. Furthermore, in regard to sleep logs, the use of self-reporting can be considered a limitation, as it introduces error in the accuracy of reporting sleep. Future studies can address this by using polysomnography, actigraphy, or apps to track sleep more accurately. Lastly, our analysis of salivary IL-6 presents two limitations. Firstly, salivary and blood levels of IL-6 are not highly correlated, and blood IL-6 may be a better indicator for exercise-induced stress, whereas salivary IL-6 is also representative of psychological stress [41]. Secondly, several values for IL-6 were above the absorbance limit of our spectrophotometer, and the repeat analysis of diluted samples was not comparable to first-thaw samples, indicating degradation during the additional freeze/thaw cycle. As such, several participants with extremely high levels of IL-6 were excluded from the analysis, decreasing the sensitivity to detect significant differences. Lastly, the inclusion of a post-training time-point for the evaluation of salivary IL-6 would have enhanced our ability to substantiate the chronic stress response during the entire semester of early-morning PT.

## 5. Conclusions

Our study is the first to identify an association between social jetlag and musculoskeletal injury, and to find that injured cadets undergo more SJL. These findings support the hypothesis that cadets with high levels of SJL may be at elevated risk of injury during time-mandated early-morning PT, which requires further evaluation, potentially in larger cohorts, in the future. Furthermore, chronotype and SJL both have a negative effect on athletic performance, as indicated by APFT scores. It is not possible to determine in the current study if poor APFT performance was the result of deficits in exercise capacity in the early morning for E-type cadets and those with SJL. Subsequent studies are required to evaluate the effects of exercise training in the context of circadian rhythm disruption (i.e., misaligned with chronotype, or chronic SJL). These metrics (chronotype, SJL) can be easily evaluated via short questionnaires, and may represent a novel tool to implement in ROTC cohorts or sports teams undergoing time-mandated training, to identify participants at higher risk of injury or compromised performance. This could inform behavioral interventions (i.e., sleep hygiene), or team/athlete decisions (i.e., practice times) at an early point of intervention to mitigate the impact of MSKI in these populations. Future studies should include an assessment of additional pro- and anti-inflammatory cytokines to more thoroughly profile the response to time-mandated exercise training. 

## Figures and Tables

**Table 1:** Anthropometric data of ROTC cohort (mean ± STD).

Variable	Value
M/F	21/6
Height (cm)	171.7 ± 10.5
Weight (kg)	75.7 ± 15.6
Age (years)	21.3 ± 3.7
BMI	25.5 ± 3.7

## Data Availability

Data are available upon reasonable request.
